# Uropathogenic *Escherichia coli* Induces Serum Amyloid A in Mice following Urinary Tract and Systemic Inoculation

**DOI:** 10.1371/journal.pone.0032933

**Published:** 2012-03-12

**Authors:** Andreja Erman, Katja Lakota, Katjusa Mrak-Poljsak, Matthew G. Blango, Veronika Krizan-Hergouth, Matthew A. Mulvey, Snezna Sodin-Semrl, Peter Veranic

**Affiliations:** 1 Faculty of Medicine, Institute of Cell Biology, University of Ljubljana, Ljubljana, Slovenia; 2 Department of Rheumatology, University Medical Centre-Ljubljana, Ljubljana, Slovenia; 3 Division of Microbiology and Immunology, Department of Pathology, University of Utah, Salt Lake City, Utah, United States of America; 4 Faculty of Medicine, Institute of Microbiology and Immunology, University of Ljubljana, Ljubljana, Slovenia; Columbia University, United States of America

## Abstract

Serum amyloid A (SAA) is an acute phase protein involved in the homeostasis of inflammatory responses and appears to be a vital host defense component with protective anti-infective properties. SAA expression remains poorly defined in many tissues, including the urinary tract which often faces bacterial challenge. Urinary tract infections (UTIs) are usually caused by strains of uropathogenic *Escherichia coli* (UPEC) and frequently occur among otherwise healthy individuals, many of whom experience bouts of recurrent and relapsing infections despite the use of antibiotics. To date, whether SAA is present in the infected urothelium and whether or not the induction of SAA can protect the host against UPEC is unclear. Here we show, using mouse models coupled with immunofluorescence microscopy and quantitative RT-PCR, that delivery of UPEC either directly into the urinary tract via catheterization or systemically via intraperitoneal injection triggers the expression of SAA. As measured by ELISA, serum levels of SAA1/2 were also transiently elevated in response to UTI, but circulating SAA3 levels were only up-regulated substantially following intraperitoneal inoculation of UPEC. In *in vitro* assays, physiological relevant levels of SAA1/2 did not affect the growth or viability of UPEC, but were able to block biofilm formation by the uropathogens. We suggest that SAA functions as a critical host defense against UTIs, preventing the formation of biofilms both upon and within the urothelium and possibly providing clinicians with a sensitive serological marker for UTI.

## Introduction

Upon entering the urinary tract, strains of uropathogenic *Escherichia coli* (UPEC) face a barage of both constitutive and inducible host defenses. Despite this hostile environment, which often includes the presence of antibiotics, UPEC is frequently able to establish itself within the host, multiply, and persist for many days to months. Persistence of UPEC within the bladder is in part attributable to the ability of these pathogens to invade urothelial cells where they can either multiply, forming large biofilm-like communities, or alternatively establish latent resevoirs which may ultimately lead to episodes of recurrent or relapsing urinary tract infections (UTIs) [1], [2]. Persistent and recurring UTIs due to UPEC represent a major clinical and financial burden worldwide [1]. The development of better approaches for the prevention and treatment of UTIs is dependent upon improved understanding of UPEC virulence strategies as well as the make-up and limitations of the host defenses employed against UPEC.

Serum amyloid A (SAA) is an acute phase protein thought to be involved in inflammation and homeostasis. SAA is a seemingly critical host defense component and has been reported to have beneficial properties in the protection against fungal, viral, and bacterial infections [3], [4], [5], [6], [7], [8], [9], and may help reduce the incidence of recurrent infections [4]. In planar lipid bilayers, SAA can assemble into hexameric structures, each containing a central channel of about 2.5 nm in diameter, and having the potential to damage bacterial membranes [10], [11]. Overexpression of SAA by intestinal epithelial cells in culture can reduce *E. coli* viability [12], and in *in vitro* assays SAA can act as an opsonin for various Gram-negative bacteria by binding to the highly expressed outer membrane protein OmpA [9]. Of note, the ability of some *E. coli* strains to invade host cells and form biofilms is critically dependent upon OmpA [13], [14], [15], [16]. Preferential binding of SAA to OmpA over endogenous host ligands, including high density lipoprotein (HDL) [4], may significantly impact both the host environment and pathogen fitness during the course of an infection.

During acute phase responses to infection, SAA secretion by hepatocytes can be greatly increased, leading to highly elevated concentrations of SAA in the circulation. This phenomenon is common among vertebrate species. For example, LPS treatment stimulates SAA expression by salmon hepatocytes [17] and in zebrafish infection models, SAA is often among the most highly upregulated gene products [18], [19], [20], [21]. In reindeer, as well as mice and humans, SAA can be used as a sensitive marker of the acute phase response to bacterial infection [22]. Similarities in SAA as an acute-phase marker in both mice and humans make mice an accessible model for investigating the regulatory and functional roles of SAA. This has not been the case with the classical human inflammatory marker C-reactive protein (CRP), which is not expressed in mice. It was recently reported, based on measurements from 219 blood donors, that SAA concentrations in sera have a median value of 20 µg/ml as determined by ELISA [23]. SAA is induced in murine models of both *E. coli*-associated mastitis [24] and colitis [12], [25], but there is currently no data regarding changes in localized or systemic levels of SAA during a UTI.

Four functional isoforms of SAA are present in mice [26]. SAA1 and SAA2 are highly homologous isotypes produced primarily in the liver. SAA3 is evolutionarily distinct from SAA1/2 and can be synthesized extrahepatically, with abundant expression found in adipose tissue and the mouse colon [12], [25]. SAA3 has also been identified as the most up-regulated message following *E. coli* infection of primary mammary epithelial cells [27]. In addition, *in vitro* experiments showed that a 10-mer peptide derived from human mammary-associated serum amyloid A3 (M-SAA3) can interfere with the adherence of enteropathogenic *E. coli* (EPEC) to intestinal epithelial cells [28]. Results presented here demonstrate that SAA1/2, and to a lesser extent SAA3, are induced both locally within the bladder and systemically in response to infection with UPEC. Furthermore, we show that UPEC is resistant to any bactericidal or bacteriostatic effects associated with SAA, although this acute phase protein can effectively inhibit biofilm formation by the uropathogens.

## Materials and Methods

### Ethics Statement

Mice were used for this study in accordance with European guidelines and the Slovenian legislation. The study was approved by the Veterinary Board of Slovenia Ethics Committee with the permit number 34401-8/2009/6.

### Animals and bacterial strains

Adult 8-week-old female C57BL/6JOLaHsd mice were housed at room temperature (22–24°C) with 45–65% humidity and a 12-hr light/12-hr dark cycle and received food and water *ad libitum*. Animals were euthanized by CO_2_ inhalation. The K-12 strain MG1655 and the UPEC isolates UTI89 and F11 have been described previously [29], [30], [31].

### SAA

Lyophilized human recombinant serum amyloid A (SAA, endotoxin-tested) (Peprotech, London, UK) was spun down, reconstituted according to manufacturer's instructions in cell culture grade sterile water to a stock concentration of 1 µg/µl, and stored at −20°C until used. This recombinant SAA is a hybrid between SAA1 and SAA2, being identical to human SAA1α except for the presence of an N-terminal methionine and SAA2β-like substitutions of asparagine to aspartic acid and arginine to histidine at positions 60 and 71, respectively.

### Mouse UTI model

Mice were anesthetized with ketamine HCl (100 mg/kg i.p.) and xylazine (10 mg/kg i.p.) prior to inoculation with a 100 µl suspension of UTI89 (10^8^ CFU) in phosphate-buffered saline (PBS) via transurethral catheterization using a sterile polyethylene catheter with 0.28 mm inside diameter (Intramedic, Becton Dickinson, Sparks, MD, USA). Before catheterization, the bladder of each animal was emptied by gentle pressure on the abdomen. Infusions were performed gradually and at a slow rate to avoid injury or vesicoureteral reflux. The catheter, sheathed over a 30 G needle connected to a 1 ml syringe, was retained in the urinary bladder for 30 min. After removal of the catheter, mice were allowed to void normally. To check if this infection protocol resulted in pyelonephritis, we tested the kidneys of infected mice. Paraffin sections of kidneys, which were recovered 24 h, 4 days and 14 days after instillation of UPEC were analyzed by a pathologist, provided no evidence of bacterial infection or inflammation. Urinary bladders, liver and blood were taken immediately after sacrifice.

### Intraperitoneal infections

Four mice were inoculated via intraperitoneal injection with 200 µl of a suspension containing 10^8^ CFU of UTI89 in PBS. The same amount of sterile PBS was received by four sham inoculated animals. After 1 day, mice were sacrificed and bladders, liver and blood were taken immediately.

### Immunofluorescence labeling

Bladders were fixed for 2 h using 3% paraformaldehyde in PBS, rinsed overnight in 30% saharose, frozen, and cut into 7-µm-thick sections. After washing in PBS, sections were permeabilized in cold acetone for 5 min, quick-dried, blocked in 3% BSA in PBS and incubated overnight at 4°C with primary antibodies against SAA (rabbit polyclonal antibodies developed against synthetically produced peptides of SAA1/2; a gift of Prof. Malle, Medical University of Graz) diluted 1∶100 in 1% BSA in PBS. After washing in PBS, samples were incubated in the dark at 37°C for 1 h with Alexa Fluor® 555 labelled goat anti-rabbit secondary antibodies (Invitrogen, Molecular Probes, Leiden, Netherlands) diluted 1∶300 in 1% BSA in PBS. After prolonged washing in PBS, sections were incubated at 37°C for 1 h with fluorescein isothiocyanate (FITC)-labelled rabbit polyclonal antibodies against *E. coli* (1∶10; Abcam, Cambridge, UK). After additional washing in PBS, sections were mounted in antibleaching mounting medium Vectashield containing 4′, 6-diamidino-2-phenylindole (DAPI) (Vector Laboratories, Burlingame, CA, USA) to stain DNA. Proper negative controls, in which both primary antibodies were replaced with serum from non-immunized animals, were performed. The samples were analysed using a Nikon Eclipse TE 300 fluorescence microscope (Amstelveen, Netherlands).

### ELISA

Mouse serum was centrifuged from whole blood collected using a syringe in the heart, aliquoted, and stored at −20°C until used. The concentrations of SAA1 (Invitrogen, CA, USA) and SAA3 (Millipore, MA, USA) were measured in duplicate assays following the manufacturer's instructions. Samples were thawed, allowed to reach room temperature and, if necessary, diluted with standard diluent buffer (1∶40 for SAA1, undiluted for SAA3, except in samples from IP injected mice, where an 1∶2000 was used) provided in the ELISA kits. After obtaining absorbance readings, SAA concentrations were calculated from standard curves. Statistical analysis was performed using mean ± SD and Student's t-tests.

### RNA isolation and quantitative PCR analysis

Total RNA from various mouse tissues, including the liver, urothelium (mechanically peeled away from the bladder), and urinary bladder wall without the urothelium, was isolated using PureLink RNA Mini Kit (Invitrogen, CA, USA) following the manufacturer's instructions. The purity and amount of RNA was determined by measuring the OD at a ratio of 260 to 280 nm. cDNA was generated from 1 µg of total RNA using the Reverse Transcription System (Promega, WI, USA) with oligo(dT) primers, and qPCR was performed with StepOne (Applied Biosystems, CA, USA) and Power SYBR Green PCR Master Mix (Applied Biosystems, Warrington, UK) using SAA-specific primers and ribosomal protein L32 primers serving as endogenous control for normalization as described in [25]. Dissociation curves of products showed only one peak in each PCR reaction. 900 nM of forward and reverse primers were used to amplify SAA1/2 and SAA3, while 300 nM of primers were used for L32. Data analysis was done using 2ΔΔCt method with normalization to the level of L32.

### Biofilm and Bacterial Growth Assays

Microtiter plate-based biofilm assays were carried out as previously described [2]. In brief, bacterial strains from overnight shaking cultures were diluted 1∶100 in M9 minimal medium, and quadruplicate 100-µl samples were incubated ±0, 0.5, 1 and 5 µg/ml SAA in 96-well pinchbar flat-bottomed polystyrene microtiter plates with lids for two days at 30°C without shaking. Nonadherent bacterial cells were then removed by gentle washings with distilled H_2_O and the remaining biofilm-associated bacteria were stained for 10 min at room temperature with 150 µl of a 0.1% solution of crystal violet in water, (Sigma-Aldrich, MO, USA). Wells were then washed with H_2_O, air dried for 30 min, and incubated for 15 min with 200 µl dimethyl sulfoxide (DMSO) to release the incorporated dye. After transferring a 150-µl aliquot from each well to a new microtiter plate, A_562_ was measured using a Synergy HT multidetection plate reader (BioTek Instruments, Inc., VT, USA).

Bacterial growth curves were acquired using quadruplicate 200-µl cultures of MG1655, UTI89, and F11 shaking in M9 minimal medium ±0, 5, or 20 µg/ml SAA at 37°C in 100-well honeycomb plates using a Bioscreen C instrument (Growth Curves, USA), as previosuly described [32].

## Results

**Figure 1:**
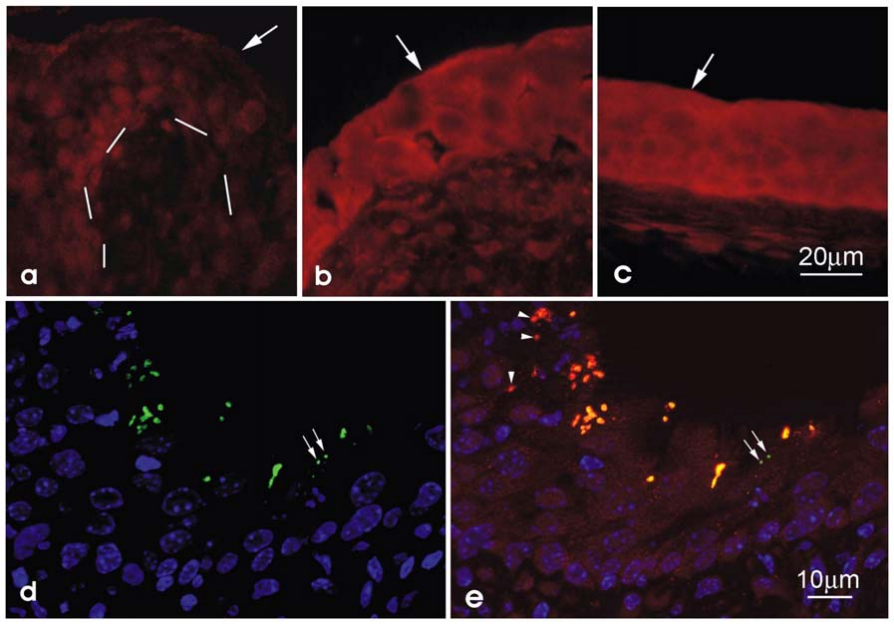
Induction and localization of SAA1/2 following inoculation of the bladder with UPEC. Immunofluorescence miscroscopy of bladder sections showing SAA1/2 (red) levels and distribution in sham-infected (PBS-treated) mice (A) and mice that were infected for either one (B) or four days (C) via intravesical instillation of the UPEC isolate UTI89. (D and E) Localization of UPEC (green) with host cell nuclei (blue) and SAA1/2 at 4 d post - inoculation of the bladder with UTI89. Large arrows point to the apical membrane of urothelium and bars mark the border between urothelium and lamina propria. Small arrows indicate a few bacteria that do not co-localize with SAA1/2.

**Figure 2:**
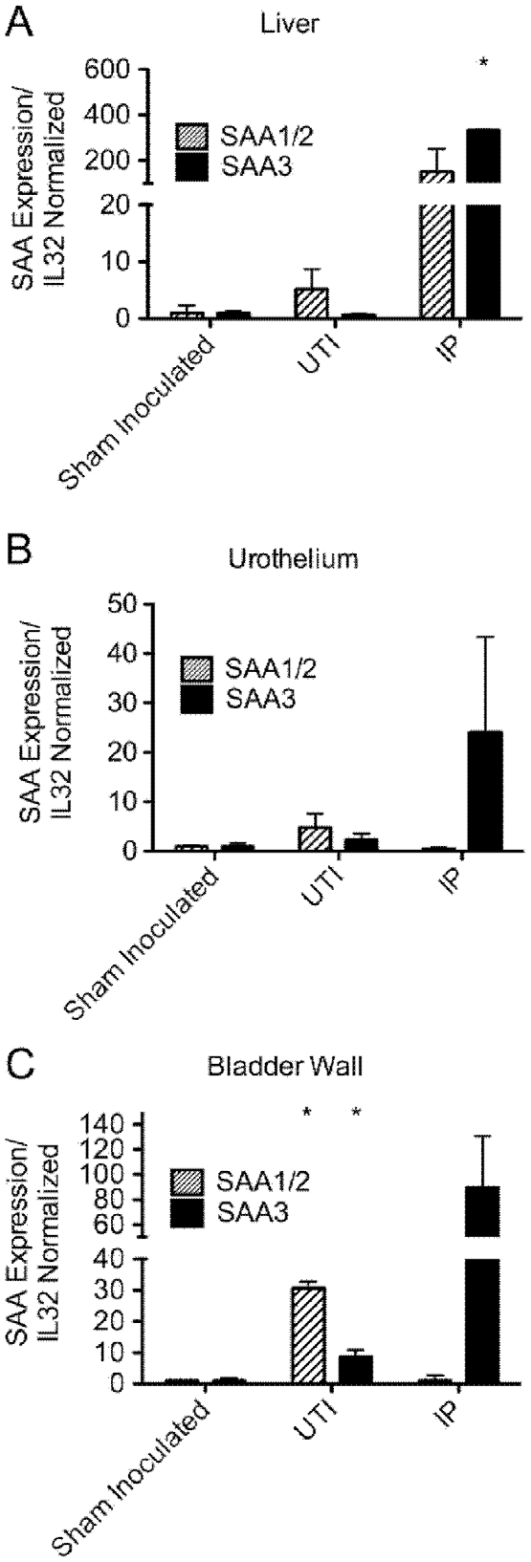
Quantification of SAA mRNA expression in response to UPEC. Four mice were either sham inoculated with PBS alone or infected with UTI89 via catheterization to initiate UTI. Alternatively, mice were infected systemically via IP injections of UTI89, with control animals recieving only PBS. After 1 d, levels of SAA1/2 and SAA3 in the (A) bladder wall, (B) isolated urothelium, and (C) liver were quantified relative to ribosomal L32 transcript levels. *, *p*<0.05 relative to sham inoculated animals as determined by Student's *t* test. The mean +/− SD of triplicate experiments are shown.

To investigate the ability of UPEC to affect SAA expression in the host, we employed a well-established mouse UTI model system. At 1 and 4 d following catheterization of adult female C57BL/6JOLaHsd mice with the UPEC isolate UTI89, levels of SAA1/2 in the urothelium were observed to be notably elevated relative to uninfected controls (**Fig. 1A–C**). Within the urothelial cells, SAA1/2 was primarily situated within the cytosol, where it co-localized strongly with internalized UPEC (**Fig. 1D and E**). Upregulation of SAA at day 1 post-inoculation of the bladder with UTI89 was confirmed by qRT-PCR of the bladder wall as well as the isolated urothelium and the liver (**Fig. 2**). SAA1/2 expression levels were elevated more than 4.8-fold in the liver and urothelium, and more than 30-fold in the bladder wall, while the relative induction of SAA3 expression in these tissues in response to UTI was less robust. However, in control experiments in which mice were infected systemically with UTI89 delivered via intraperitoneal (IP) injection, SAA3 expression was markedly increased in the liver (more than 300-fold) as well as in the urothelium (∼20-fold) and bladder wall (∼90-fold).

**Figure 3:**
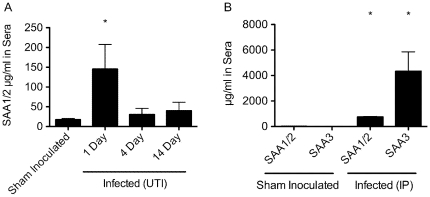
ELISA of mouse sera at different time points after infection. Four mice were inoculated with UTI89 via either (A) transurethral catheterization or (B) IP injection. (A) Serum levels of SAA1/2 and SAA3 in mice were quantified by ELISA in sham inoculated animals and at 1, 4, and 14 d after UTI89 instillation into the bladder (UTI). SAA3 was not detected in these samples. (B) Substantially higher serum levels of SAA1/2 and SAA3 were detected 1 d after infection with UTI89 via IP injection. *, *p*<0.05 relative to sham inoculated animals as determined by Student's *t* test. The mean +/− SD of triplicate experiments are shown.

Within 1 d following instillation of mouse bladders with UTI89, we observed by ELISA a substantial 8-fold increase in SAA1 serum levels, which decreased by day 4 and remained subdued up to day 14 (**Fig. 3A**). In contrast, serum levels of SAA3 were not altered in response to UTI (data not shown). However, following IP injection of UTI89 we did detect significant and notably large increases in circulating SAA3 levels along with corresponding, although smaller, increases in SAA1/2 levels (**Fig. 3B**). This is the first time, to our knowledge, that serum SAA3 protein levels have been shown to increase substantially in response to UPEC delivered intraperitoneally.

**Figure 4:**
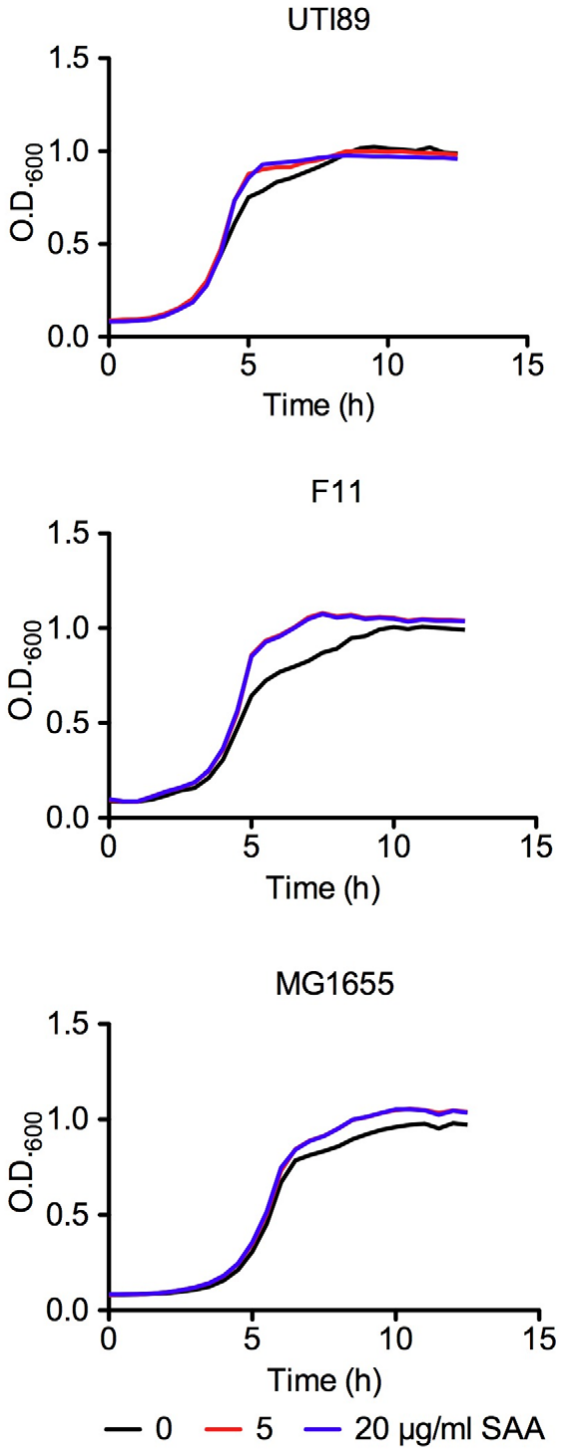
Growth of UTI89, F11, and MG1655 ± SAA. Bacterial growth in M9 medium with 0, 5, or 20 µg/ml SAA was determined by measuring OD_600_ over time. Each curve represents the means of results from quadruplicate samples.

**Figure 5:**
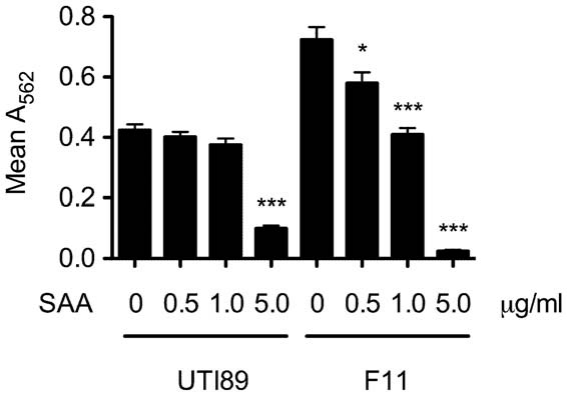
Biofilm formation by the UPEC isolates UTI89 and F11 ± SAA. Biofilm levels were quantified by measuring A_562_ after 48 h static growth in M9 medium ±0, 0.5, 1 and 5 µg/ml SAA. *, *p*<0.001, as determined by Student's *t* test. Results show the mean values ± SEM of three independent experiments performed in quadruplicate.

Previous work indicated that recombinant SAA1/2 expressed by cultured host cell lines can inhibit the growth and viability of laboratory *E. coli* K-12 strains [12], raising the possibility that SAA may act similarly against UPEC isolates during the course of a UTI. However, in *in vitro* assays, we found that growth of the UPEC isolates UTI89 and F11 in M9 medium was unfazed by SAA concentrations up to 100 µg/ml (data not shown and **Fig. 4**), indicating that SAA does not have direct bactericidal or bacteriostatic effects on UPEC. Interestingly, SAA also had no effect on growth of the reference K-12 isolate MG1655 in our assays. In considering alternate means by which SAA could potentially affect the pathogenesis of UTIs, we noted previous reports indicating that SAA can opsonize Gram-negative bacteria by binding the abundant outer membrane protein OmpA [4], [9]. OmpA is an important facilitator of biofilm formation by UPEC and other *E. coli* isolates (unpublished observations and [15]), and biofilm formation both on and within the urothelium is thought to promote the establishment and persistence of UPEC within the urinary tract [1], [30]. To test possible effects of SAA on biofilm formation by UPEC, we employed standard *in vitro* microtiter plate biofilm assays. In these assays, the addition of a physiological concentration of SAA (5 ug/ml) significantly inhibited biofilm formation by UTI89 as well as F11 (**Fig. 5**).

## Discussion

A few decades ago the first publication emerged indicating that SAA and CRP act as the most reliable markers for monitoring antimicrobial therapy in patients with UTIs [33]. SAA and CRP levels were also found to be higher in bacterial infections than in viral infections, although SAA appeared to be more clinically relevant as a marker of inflammation in acute viral infections [34]. Initial reports on the anti-microbial activities of SAA were published about a decade ago with work showing that recombinant SAA can enhance the anti-fungal activities of polymorphonuclear cells (PMNs) [35]. Within 30 min of stimulation, SAA was found to promote the upregulation of cytosolic Ca^++^ concentrations, cell-surface expression of CD11c and CD16 (antigens involved in adhesion and microbial recognition), and elevation of lactoferrin secretion. Lactoferrin itself is an anti-microbial agent, which enchances PMN phagocytic activity against heat-killed yeast *Candida albicans*.

More recently, SAA was shown to interact with hepatitis C (HCV) virions, thereby blocking viral entry [6]. Although no clinical correlation in this study could be found between sera levels of SAA and HCV viral loads, a tight relationship between SAA and HDL levels in modulating HCV infectivity has been proposed [3], [5]. SAA has also been shown to inhibit HIV-1 infection of host cells via CCR5 receptors [5], [7]. SAA appears to be one of the earliest systemic anti-viral responses to HIV-1, being induced as early as 5–7 days prior to the first detection of plasma viral RNA and considerably earlier than other systemic cytokines. Such observations indicate that SAA can act as a front line anti-viral defense prior to systemic activation of other immune responses [5].

SAA also appears to play a major role as host defense against bacterial pathogens, acting as an opsonin able to rapidly bind with high affinity to many Gram-negative bacteria, including *E. coli*, *Pseudomonas aeruginosa*, *Salmonella typhimurium*, *Shigella flexneri*, *Klebsiella pneumoniae*, and *Vibrio cholerae* [4]. Binding of SAA to *E. coli* was found to be mediated primarily through interactions with OmpA, as OmpA deficient *E. coli* did not to bind SAA [9]. Importantly, the concentration of SAA required for half-maximal binding to *E. coli* is within physiologically relevant levels and does not require other acute phase responses [4]. *In vitro*, neutrophils phagocytose K-12 *E. coli* more readily and in higher numbers following opsonization of the bacteria with physiologically normal levels of SAA [9]. More recently, overexpression of SAA1/2 by epithelial cells has been shown to reduce the viability of a co-culured K-12 *E. coli* strain via an as-yet undefined mechanism [12]. In contrast to these antimicrobial effects, SAA may in some cases be detrimental to the host. For example, SAA has been shown to inhibit local inflammatory responses to *Actinetobacter baumannii Pneumonia* (a Gram-negative pathogen that is often resistant to many antibiotics), and may thereby actually facilitate, rather than inhibit, bacterial survival and outgrowth [8].

Here we report for the first time that SAA (specifically SAA1/2 and to a smaller extent SAA3) is greatly induced in the bladder wall and, to a lesser extent, the urothelium in response to UPEC instilled into the urinary tract via catheterization. As seen by immunofluorescence microscopy, infection with UPEC resulted in high levels of cytoplasmic SAA in comparison to the more subdued, primarily nuclear expression pattern of SAA in uninfected urothelial cells (see Fig. 1). Heightened levels of SAA expression within the bladder wall versus the urothelium suggest that infiltrating immune effector cells and resident host cells within this compartment are primary contributors to SAA production during a UTI. Enhanced levels of SAA1 expression in response to UPEC within the urinary tract were also observed systemically, being detected in the liver and transiently within the serum of infected mice. Direct inoculation of UPEC into the peritoneum also increased levels of SAA1 and SAA3 within both the liver and general circulation, with only SAA3 increased in the bladder wall and urothelium.

Although the physiological role of SAA during a UTI remains to be tested *in vivo*, the robust localized and systemic amplification of SAA in response to infection with UPEC suggests a critical role for this acute phase protein as a host defense against UTIs. UPEC isolates are often more resistant to environmental stresses and host defense mechanisms [36], and this phenomenon appears to also be true in the case of SAA. While SAA expressed by cultured host cells can interfere with the viability of laboratory *E. coli* K-12 strains [12], we found that growth of UPEC as well as the K-12 strain MG1655 proceeds unhindered even in the presence of high levels of SAA. It may be that the previously reported bacteridal effects of SAA require additional co-factors not present in our *in vitro* assays. However, we did find that SAA can potently inhibit the ability of UPEC isolates to form biofilms, possibly by binding to and occluding OmpA. The formation of both extra- and intracellular biofilm communities by UPEC can impact the establishment and persistence of these microbes within the urinary tract [13], [32], [37]. By interfering with biofilm development, SAA may increase the susceptibility of UPEC to other host defense mechanisms while also depriving the pathogens of a strong foothold within the urothelium in which to expand their numbers.
